# Dataset showing thermal conductivity of South-Eastern Nigerian kaolinite clay admixtures with sawdust and iron filings for fired-bricks production

**DOI:** 10.1016/j.dib.2019.104708

**Published:** 2019-11-06

**Authors:** Chigbo A. Mgbemene, Esther T. Akinlabi, Omolayo M. Ikumapayi

**Affiliations:** a Department of Mechanical Engineering, University of Nigeria, 41001, Nsukka, Nigeria; b Department of Mechanical Engineering Science, University of Johannesburg, 2006, South Africa; c Department of Mechanical Engineering, Covenant University, Ota, Nigeria

**Keywords:** Additives, Iron filings, Kaolinite clay, Sawdust, Thermal conductivity

## Abstract

In this dataset, the influence of admixture of sawdust and iron filings on the kaolinite clay was experimented. This was done by blending various samples of kaolinite clay with varying percentages of sawdust and iron filings. Thermal analysis of the clay samples was carried out at different ratios of sawdust and iron filings blended with the clay samples. The blended ratio of sawdust and iron fillings ranges from 0%, 5%, 10%, 20%, 30%–40%. These samples were fired in a local kiln that achieved temperature of 900 °C - 1200 °C to burn-off the sawdust consequently creating pores/cavities where the sawdust had been burnt and to fuse the iron particles with the clay material. The experimental data on the thermal characteristics and refractory properties of the clay sample were then acquired. The data were acquired, processed and presented. Thermocouple and thermometer were used to acquire the temperature during the firing of the bricks. Finally, thermal conductivities and bulk densities of the samples were computed following an established standard.

**Table:** Specifications Table

Subject area	*Mechanical and Production Engineering*
More specific subject area	*Geothermal and Structural Engineering*
Type of data	*Tables, and Figures*
How data was acquired	*Field survey, sample collection (Kaolinite Clay, Sawdust and Iron Filings), preparation (admixtures, firing, thermocouple and thermometer measurements) and laboratory analysis*
Data format	*Raw data collection and Analysis*
Experimental factors	*The clay sample was soaked in water and impurities were removed. It was then dried, crushed and sorted. Foreign bodies and non-clay lumps were removed. The sorted particles were then mixed with the smaller sized clay and all were further crushed into very fine particle sizes and sieved with* 250 μm *and* 425 μm *mesh sieves. The* 250 μm *sample was used for the chemical analysis while the* 425 μm *sample was used for brick production. The first portion was mixed with sawdust of average particle size of 0.*1 mm *in the following percentage by volume 5%, 10%, 20%, 30%, and 40%. The second portion was mixed with iron filings of average particle size of 0.*3 mm *in the same order of percentage. The third portion had 10% sawdust and varying percentages of iron filings in the same increasing order of percentage. The fourth portion had 20% sawdust and the same increasing order of percentage of iron filings. The last portion had 30% sawdust and like the rest, iron filings in the same increasing order of percentage. Each portion was then thoroughly mixed; then molded damp with about 10% water content into rectangular bricks. The bricks were then kept in the sun for 72 hours to dry out. But to be sure the samples dried out properly, they were placed in an oven at about 100*^*o*^*C for 3 hours. After that, the samples were fired in a local kiln that achieved temperatures of 900*^*o*^*C ∼1200*^*o*^*C*
Experimental features	*The clay sample was determined to belong to the kaolinite group. The iron filings with an average particle size of 0.*3mm *were obtained from the Mechanical Engineering Department's laboratory. The sawdust was obtained from the Civil Engineering Department's laboratory. The sawdust was mostly of Mahogany wood. The geometry of the sawdust was varied with the average particle size standing at about* 0.1mm.
Data source location	*New Layout-Enugu at Latitudes 6°30′N and Longitudes 7°30′E, Enugu State in Eastern Nigeria. The deposit site was situated about* 60 km *from the University of Nigeria, Nsukka where the experiment was carried out.*
Data accessibility Related research article	*The availability of the dataset is within the peripheral of this article* *Mgbemene CA, Akinlabi ET, and Ikumapayi OM. Influence of additives on kaolinite clay properties – applications, trends, and a case study. Procedia Manufacturing 35(2019) 1395 – 1399. 10.1016/j.promfg.2019.09.009* [1].

**Table:** 

**Value of the Data** • *The dataset is vital for researchers who are aiming at new sustainable structural and construction materials with low cost and eco-friendly that enhanced thermal conductivity for building and civil applications.* • *The reported thermal conductivity could serve as a yardstick or benchmark in the field for laboratory testing of bricks in structures.* • *The percentage of admixtures could be used to validate the modeling methods of thermal conductivity.* • *The recorded* densities *could be used to determine the integrity of structural components based on the admixtures applied. The heavier the better in terms of structure for rigidity, durability, stability solidity and firmness.*

## 1 Data

**Fig. 1:**
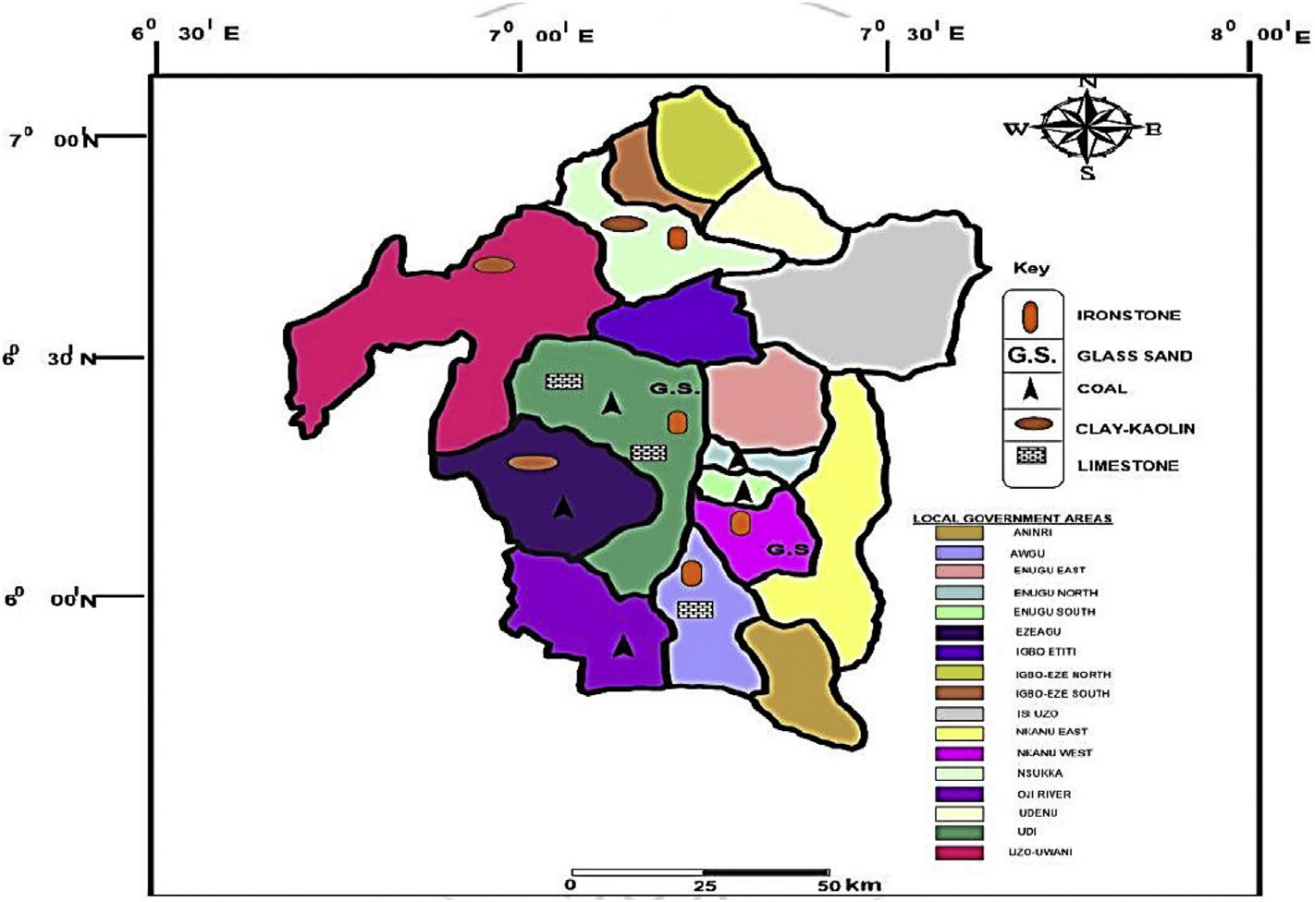
Location of Sample area showing deposition of Kaolinite Clay in the Map of Enugu.

**Fig. 2:**
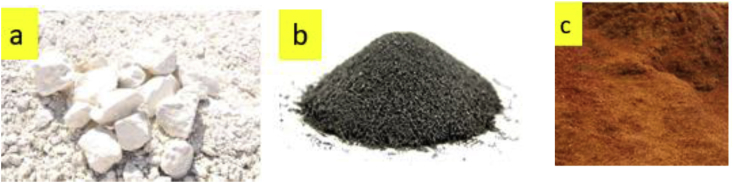
**Additives Materials** (a) Kaolinite Clay (b) Iron Filings particles (c) Sawdust particles.

**Fig. 3:**
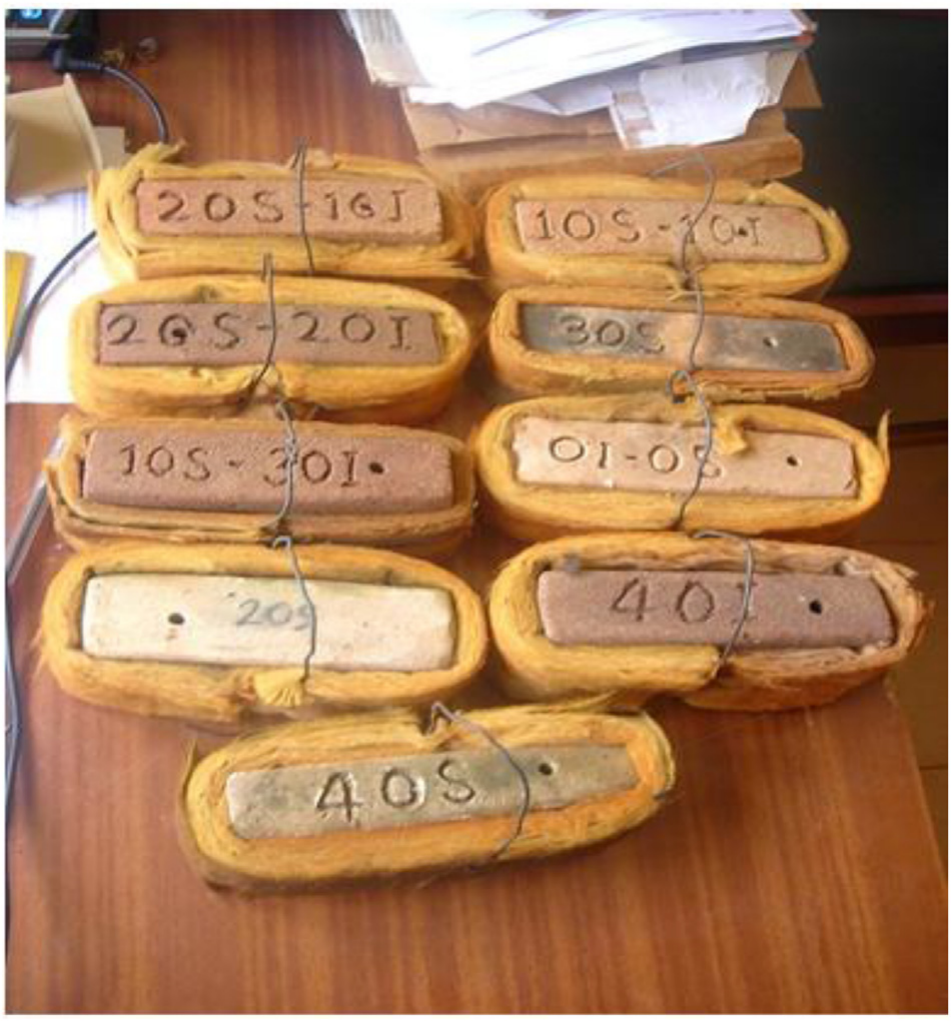
Some of the Clay samples used in the experiment.

This article presents thermal conductivity of kaolinite clay obtained from a clay deposit site in New Layout-Enugu at Latitudes 6°30′N and Longitudes 7°30′E, Enugu State in Eastern Nigeria (see Fig. 1 showing the deposition of kaolinite clay in Enugu State). The deposit site was situated about 60km from the University of Nigeria, Nsukka where the experiment was carried out. The clay sample as shown in Fig. 2a was determined to belong to the kaolinite group. The iron filings as shown in Fig. 2b with an average particle size of 0.3mm were obtained from the Mechanical Engineering Departmental laboratory. The sawdust as shown in Fig. 2c was obtained from the Civil Engineering Departmental laboratory. The sawdust was mostly of Mahogany wood. The geometry of the sawdust was varied with the average particle size standing at about 0.1mm. Some of the produced fired-bricks with admixture of sawdust and iron-filings are presented in Fig. 3 while the experimental set-up for the data acquisition is depicted in Fig. 5. The Acquired data from the experimental set-up were presented in Table 1 – 5 which contained the admixture of sawdust and iron-filings of varying percentage compositions. Fig. 6, Fig. 7, Fig. 8, Fig. 9 show the comparison plots in the thermal conductivities and densities of the produced fired-bricks at a varying percentage of reinforcements of sawdust and iron-filings.

**Table 1:** Thermal conductivities and densities dataset at varying % Sawdust.

% Sawdust	Density, ρ (kg/m^3^)	Thermal Conductivity, *k* (W/mK)
0	1812	1.65
5	1660	1.58
10	1593	1.52
20	1550	1.51
30	1404	1.49
40	1294	1.48

## 2 Experimental design, materials and methods

### 2.1 Preparation of samples and methods

The clay sample was soaked in water and impurities were removed. It was then dried and crushed. A coarse sieve of about 0.8mm was used to sieve the sample first. The big size particles were separated and sorted; foreign bodies and non-clay lumps were removed. The sorted particles were then mixed with the smaller sized clay and all were further crushed into very fine particle sizes and sieved with 250 μm and 425 μm mesh sieves. The 250 μm sample was used for the chemical analysis while the 425 μm sample was used for brick production. The 425 μm clay sample was then divided into several portions with one portion not mixed with any additives. This would serve as the standard for comparing the other samples. The other portions were then mixed with each additive to a specified percentage by volume as required. The first portion was mixed with sawdust in the following percentage by volume 5%, 10%, 20%, 30%, and 40%. The second portion was mixed with iron filings in the same order of percentage. The third portion had 10% sawdust and varying percentages of iron filings in the same increasing order of percentage. The fourth portion had 20% sawdust and the same increasing order of percentage of iron filings. The last portion had 30% sawdust and like the rest, iron filings in the same increasing order of percentage. Each portion was then thoroughly mixed; then molded damp with about 10% water content into rectangular bricks.

The bricks were then kept in the sun for 72 hours to dry out. But to be sure the samples dried out properly, they were placed in an oven at about 100 °C for 3 hours. After that, the samples were fired in a local kiln that achieved temperatures of 900 °C–1200 °C. According to Daunoravičiūtė, and Petrikaitis [2], 59.9% of sawdust burns out at 380 °C, this implies that at 900 °C all the sawdust would have burnt off leaving only voids where they had been. Finally, the samples were all weighed and the density of each one was calculated. Fig. 3 shows some of the clay brick samples used in the experiment.

The boundary conditions of the experiment were the additive size, the quantity of the additive and the firing temperature. The additive size and quantity affected the strength and thermal properties of the clay. The more the sawdust added, the lower the strength and the better the insulatory properties but subject to a point where the structural integrity of the clay material failed. Increasing the quantity and size of the iron filing increased the strength and lowered the insulatory properties of the clay. Extremes of these conditions had detrimental effects on the clay material. To avoid these, the maximum limit of the additive did not exceed 40% by weight. The firing temperature had to be kept below the vitrification temperature of clay and also was kept below the melting point of iron.

### 2.2 Determination of the thermal conductivity

**Fig. 4:**
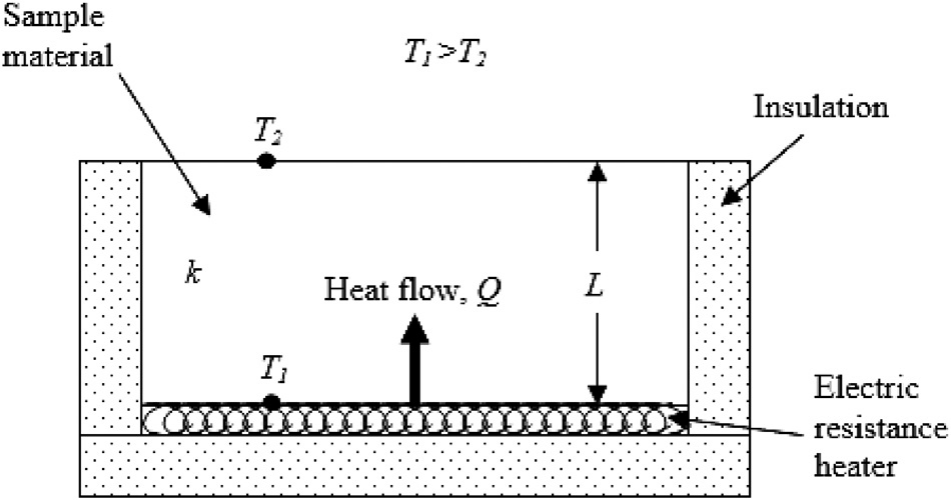
The schematic diagram of the experimental setup.

There are several setups for determining the thermal conductivity of materials [3] but for the purpose of this research work, the experimental setup adopted was from Cengel [4] and Drpic et al. [5] in which EN 1745:2002 standard was used for the determination of thermal conductivity and thermal resistance of masonry and masonry products and the schematic diagram for thermal conductivity experimental set-up is depicted in Fig. 4. According to Enibe and Iloeje [6], the two methods recommended for determining the thermal properties of soils, rocks, and mineral deposits are the thermal comparator method and the periodic method but for the simple geometry of the clay samples, the steady-state method equally gives reliable data and is also simpler to apply.

The temperature readings obtained gave an idea of the temperature distribution in each sample. When steady heat transfer was reached, which occurred after about 20 minutes, the temperatures were recorded. The thermal conductivity was computed using equations (1), (2), (3) [7].

From Fourier's law of heat conduction, the thermal conductivity, *k* is obtained as

(1) $$k=\frac{QL}{A\left(T_{1}-T_{2}\right)}$$

or

(2) $$k=\frac{Q^{\prime \prime }L}{\left(T_{1}-T_{2}\right)}$$

where *Q* = heat transfer rate [W].

*L* = thickness of brick [m].

*A* = the brick's heat transfer surface area [m^2^].

*T*_*1*_=temperature of the heat inlet surface [^o^C].

*T*_*2*_ = temperature of the heat outlet surface [^o^C].

$Q^{\prime \prime }$ = heat flux [W/m^2^]

(3) $$Q^{\prime \prime }=Q/A$$

**Fig. 5:**
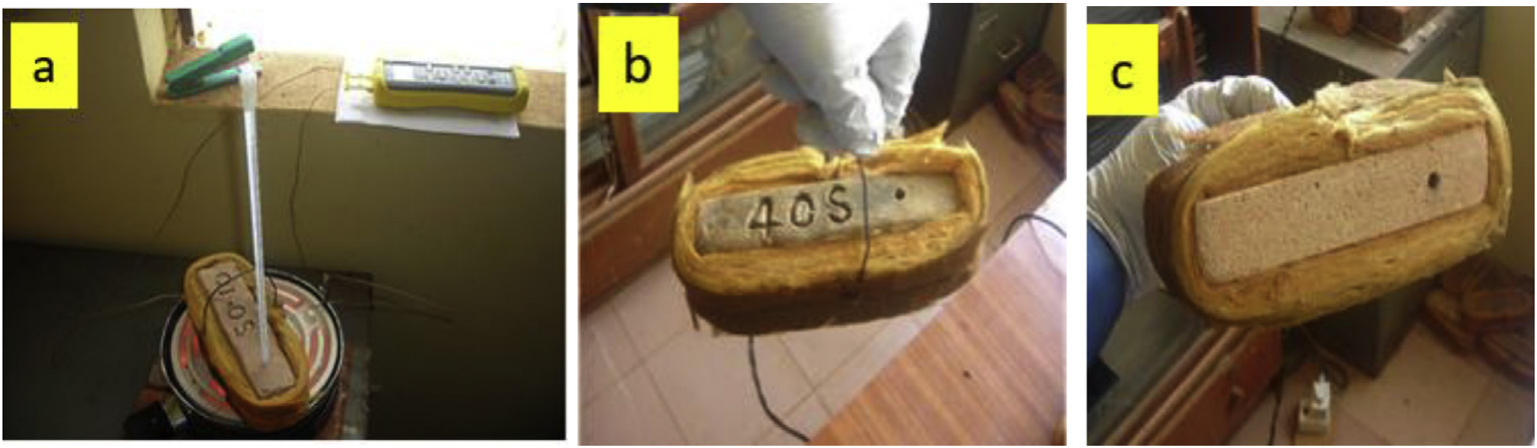
(a) The experimental set-up with a sample having the thermocouples and the thermometer all in place (b) The upper heat transfer surface of an insulated Samples (c) The lower heat transfer surface of an insulated sample.

Fig. 5a shows the setup with a sample and the measuring devices in place. The thermometer was calibrated to match the readings from the thermocouple. Each clay sample was insulated all round with glass fiber leaving only two opposite surfaces for heat transfer as shown in Fig. 5b and c. Each sample was placed with an uninsulated surface on an electric resistance heater which produced an average heat flux of 15,892W/m^2^ and heat was supplied through that surface. Two digital K-type thermocouple probes of HH 12B Omega thermocouples were placed on those two uninsulated surfaces to measure the surface temperatures. The core of each sample was bored and a thermometer was placed inside at a specified depth.

### 2.3 Acquired data

The thermal conductivities of the samples were calculated using Equation (2). The data for each sample is presented, along with their densities, in Table 1, Table 2, Table 3, Table 4, Table 5.

**Table 2:** Thermal conductivities and densities dataset at 10% Sawdust and varying % Iron- Filings.

10% Sawdust		
% Iron Filings	Density, ρ (kg/m^3^)	Thermal Conductivity, *k* (W/mK)
0	1593	1.52
5	1675	1.74
10	1755	1.81
20	1932	1.91
30	2139	2.12
40	2231	2.24

**Table 3:** Thermal conductivities and densities dataset at 20% Sawdust and varying % Iron- Filings.

20% Sawdust		
% Iron Filings	Density, ρ (kg/m^3^)	Thermal Conductivity, *k* (W/mK)
0	1550	1.51
5	1632	1.73
10	1707	1.79
20	1844	1.89
30	1968	2.04
40	2096	2.14

**Table 4:** Thermal conductivities and densities dataset at 30% Sawdust and varying % Iron- Filings.

30% Sawdust		
% Iron Filings	Density, ρ (kg/m^3^)	Thermal Conductivity, *k* (W/mK)
0	1404	1.49
5	1477	1.53
10	1553	1.6
20	1712	1.61
30	1876	1.64
40	2031	1.65

**Table 5:** Thermal conductivities and densities dataset at varying % Iron- Filings.

% Iron Filings	Density, ρ (kg/m^3^)	Thermal Conductivity, *k* (W/mK)
0	1812	1.65
5	1836	1.76
10	1891	1.82
20	2067	1.93
30	2282	2.21
40	2416	2.39

#### 2.3.1 Data nomenclatures

5I indicates the sample which has 5% iron filings, 10I indicates sample with 10% iron filings; 5S indicates the sample with 5% sawdust, 10S 5I indicates sample with 10% sawdust and 5% iron filings, etc.

The sample not mixed with any additives was designated as 0S 0I with a density of 1812 kg/m^3^ and a thermal conductivity of 1.65W/mK.

**Fig. 6:**
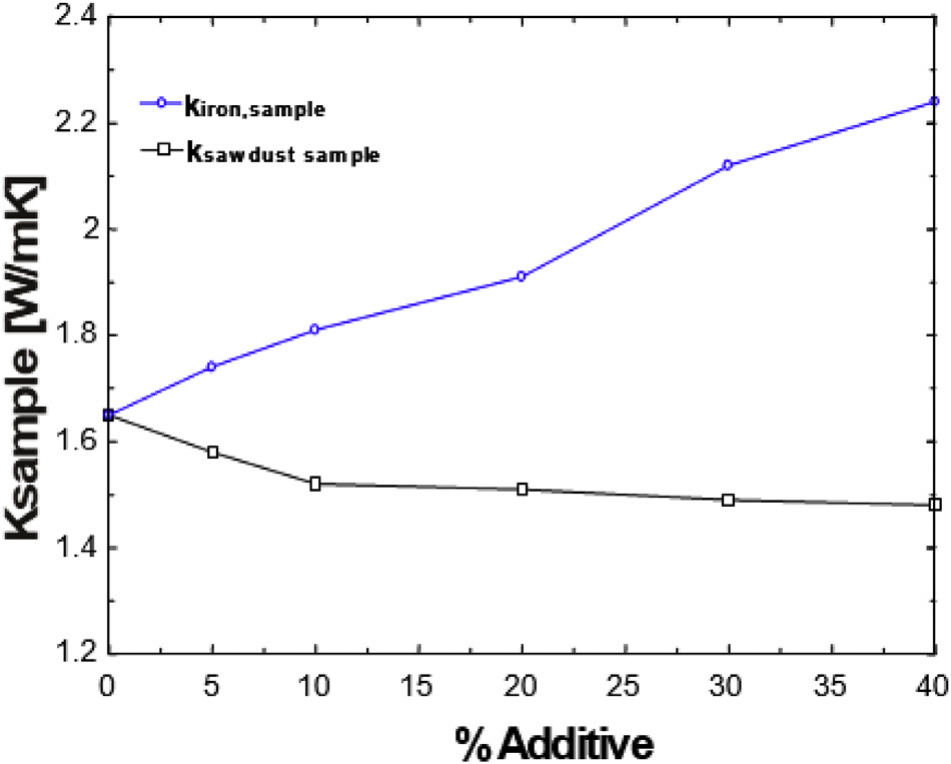
Thermal conductivity versus % additive for individual sawdust and iron filings samples.

**Fig. 7:**
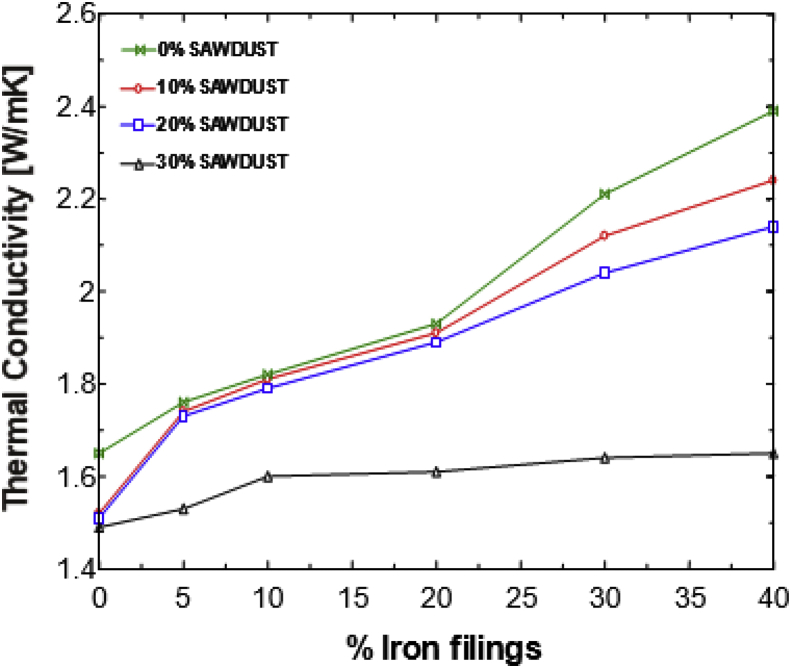
Thermal conductivity against percentage Iron filings plots for the admixture's samples.

Looking at the effect of the additives on the thermal conductivities of the clay samples, one observes that the thermal conductivity of the clay increased by the addition of iron filings while it decreased by the addition of sawdust as shown in Fig. 6. At temperatures of 900 °C and above, the iron filings melted and fused with the clay creating a tightly packed composition. The sawdust burnt off creating pores in the clay structure. When the two additives are mixed, one expects the data to be somewhere in between the two effects, but the data show that the effect of the addition of iron filings becomes more dominant in 10S as shown in Fig. 7. In the 20S, the effect is slowed down, but in 30S, the effect of the sawdust greatly becomes more dominant, suppressing the effect of the iron filings. This could be seen from the large space existing between the graphs of the 20S and 30S. This data in the thermal conductivity falling below or becoming equal to that of the clay without additives.

**Fig. 8:**
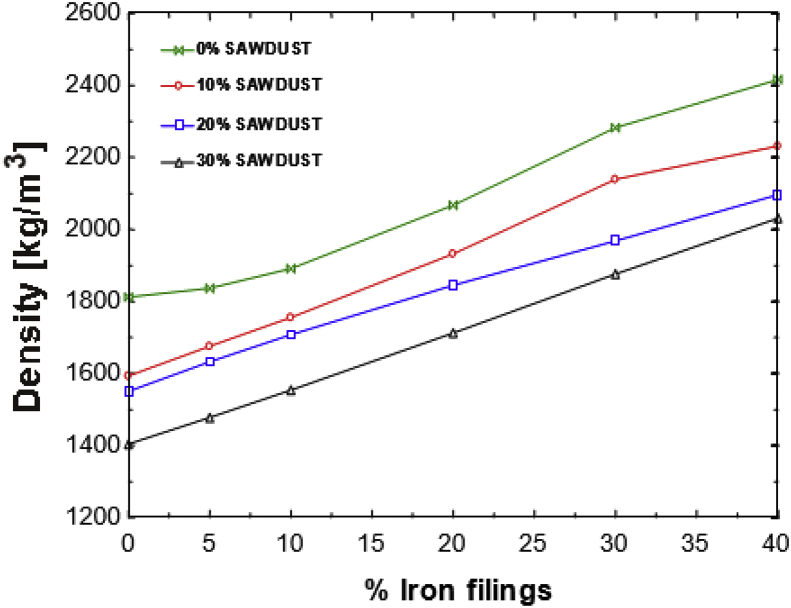
A plot of Density against percentage Iron filings for the admixture's samples.

**Fig. 9:**
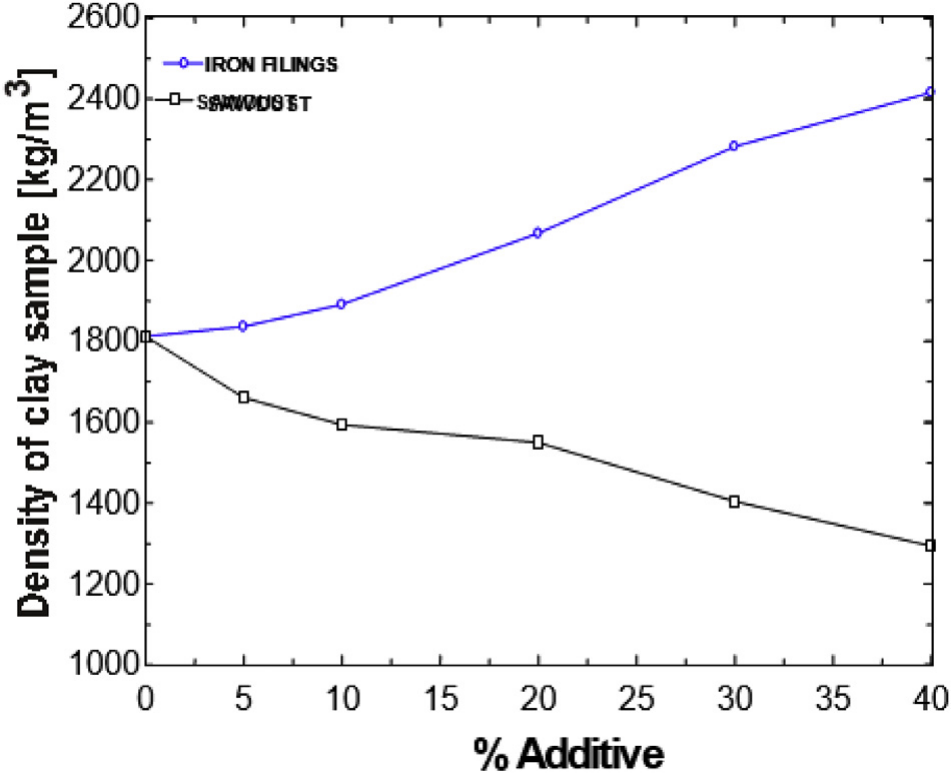
Density versus percentage additive for individual sawdust and iron filings samples.

The large difference in the thermal conductivity is curiously not present in the bulk density plots of Fig. 8 as one would expect because the densities increased proportionally with the thermal conductivities. The 30S 40I presents interesting data in which its thermal conductivity is the same as that of a clean sample but the bulk density is much higher than that of the clean sample Fig. 9. The thermal conductivity of 1.65W/mK and density of 2031kg/m^3^ fall within the range of those of fire clays but the density is high for kaolinite clay. Although the data acquired concentrated more on the thermal conductivity of the clay, it is worth noting some of the effects on some physical properties of the sample. The physical appearance of each sample was affected by the additives. The weights also varied according to the additive-the more the sawdust, the lighter the sample; the 40S sample was the lightest. Also, the more the iron content, the heavier the sample hence, the 40I sample was the heaviest. The clean sample was the smoothest while the 30S 40I sample had the roughest composition and had the least fracture strength. The effects of the additives on clay have been experimented and it was found that when two additives – sawdust and iron filings, are introduced into the clay at the same time and in various proportions, there is a possibility of developing new material with new characteristics. This is highlighted because the material with 30% sawdust and 40% iron filings has a density of 2031 kg/m^3^ which is high for kaolinite clay material and thermal conductivity of 1.65 W/mK which is normal for clay. Despite this, the material still retains the properties of fire clay. Further new materials can also be developed by varying the sawdust/iron filings percentages mix alternately [8].
